# Supplementary material for: Household Microenvironment and Under-Fives Health Outcomes in Uganda: Focusing on Multidimensional Energy Poverty and Women Empowerment Indices
Source: Int J Environ Res Public Health. 2022 May 30;19(11):6684. doi: 10.3390/ijerph19116684 (PMC9180902; doi:10.3390/ijerph19116684)
Supplement: Supplementary file 1 [file ijerph-19-06684-s001.zip › ijerph-1691180-supplementary.pdf]

**Annex:** Results from different Logit model specifications for the association between acute respiratory infection (ARI) and Multidimensional Energy Poverty Index (MEPI).

**Table S1.** The Odds ratios and confidence intervals with significance level for the studied covariates from different logistic regression model specifications for ARI

| **Variables (Base Category)** | **Odds Ratios (ORs) and 95% Confidence Intervals (CI) od Model [1] Results** | | |
|---|---|---|---|
| | **Model 1** | **Model 2** | **Model 3: Full Model** |
| **Multidimensional energy poverty (moderate)** | | | |
| Multidimensionally Acute Energy Poor | 1.68 ***(1.44 to 1.97) | 1.39 *** (1.16 to 1.66) | 1.32 *** (1.10 to 1.58) |
| **Drinking water quality (unimproved)** | | | |
| Improved | | 1.01 (0.88 to 1.17) | 0.99 (0.85 to 1.5) |
| **Sanitation facility (No facility)** | | | |
| Unimproved | | 0.51 *** (0.43 to 0.60) | 0.55 ***(0.47 to 0.65) |
| Improved | | 0.50 ***(0.41 to 0.62) | 0.55 *** (0.45 to 0.68) |
| **Hygiene facility (No facility)** | | | |
| Limited | | 0.84 ** (0.74 to 0.97) | 0.87 * (0.77 to 1.01) |
| Basic | | 0.73 ***(0.61 to 0.86) | 0.75 *** (0.63 to 0.89) |
| **Scores for the three women empowerment domains** | | | |
| Score for attitude to violence | | | 0.88 *** (0.82 to 0.93) |
| Score for social independence | | | 0.91 ** (0.84 to 0.98) |
| Score for decision making | | | 1.05 (0.98 to 1.12) |
| **Sex of child (Male)** | | | |
| Female | 0.94 (0.83 to 1.06) | 0.94 (0.84 to 1.06) | 0.93 (0.82 to 1.04) |
| **Child age category (0-11 months)** | | | |
| 12-35 months | 0.99 (0.86 to 1.16) | 0.99 (0.86 to 1.16) | 1.001 (0.86 to 1.17) |
| 36-59 months | 0.68*** (0.58 to 0.79) | 0.68 *** (0.58 to0.79) | 0.67*** (0.57 to 0.79) |
| **Place of residence (Urban)** | | | |
| Rural | | 1.17 (0.96 to 1.42) | 1.12 (0.92 to 1.36) |
| Constant | 0.09 (0.07 to 0.10) | 0.17 ***(0.13 to 0.24) | 0.17 *** (0.13 to 0.24) |
| Number of observations | 12,488 | 12,488 | 12,095 |
| $\chi^2_{df}$ $(P - value)$ | 76.82 (p < 0.01) | 176.50 (p < 0.01) | 203.81 (p < 0.01) |
| McFadden's or the Pseudo $R^2$ | 0.0104 | 0.0217 | 0.025a |

[1] Model 1-controlling individual covariates; Model 2- controlling for individual and household microenvironment related covariates; Model 3- is full model controlling for individual and household microenvironment related covariates and Women empowerment. *** $p < 0.01$, ** $p < 0.05$, * $p < 0.1$
